# Comparison of Physicochemical, Antioxidant, and Cytotoxic Properties of Caffeic Acid Conjugates

**DOI:** 10.3390/ma17112575

**Published:** 2024-05-27

**Authors:** Grzegorz Świderski, Ewelina Gołębiewska, Monika Kalinowska, Renata Świsłocka, Natalia Kowalczyk, Agata Jabłońska-Trypuć, Włodzimierz Lewandowski

**Affiliations:** Department of Chemistry, Biology and Biotechnology, Institute of Civil Engineering and Energetics, Faculty of Civil Engineering and Environmental Science, Bialystok University of Technology, Wiejska 45E Street, 15-351 Bialystok, Poland; e.golebiewska@pb.edu.pl (E.G.); m.kalinowska@pb.edu.pl (M.K.); r.swislocka@pb.edu.pl (R.Ś.); natalia.kowalczyk@sd.pb.edu.pl (N.K.); a.jablonska@pb.edu.pl (A.J.-T.); w-lewando@wp.pl (W.L.)

**Keywords:** caffeic acid conjugates, theoretical calculations, FT-IR, Raman, ^1^H and ^13^C NMR, UV-VIS, SOD activity, HO^•^, cytotoxic study (DLD-1)

## Abstract

Spectroscopic studies (FT-IR, Raman, ^1^H, and ^13^C NMR, UV-VIS) of caffeic acid (CFA) and its conjugates, i.e., caftaric acid (CTA), cichoric acid (CA), and cynarin (CY), were carried out. The antioxidant activity of these compounds was determined by a superoxide dismutase (SOD) activity assay and the hydroxyl radical (HO^•^) inhibition assay. The cytotoxicity of these compounds was performed on DLD-1 cell lines. The molecules were theoretically modeled using the B3LYP-6-311++G(d,p) method. Aromaticity indexes (HOMA, I6, BAC, Aj), HOMO and LUMO orbital energies and reactivity descriptors, NBO electron charge distribution, EPS electrostatic potential maps, and theoretical IR and NMR spectra were calculated for the optimized model systems. The structural features of these compounds were discussed in terms of their biological activities.

## 1. Introduction

**Figure 1:**
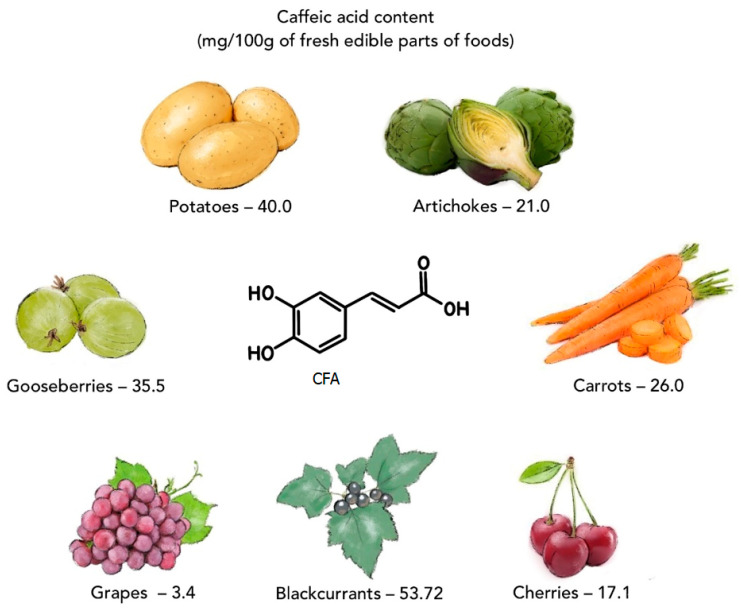
Selected plant products containing caffeic acid (on the basis of data from [5]).

Caffeic acid (CFA) is a natural phenolic compound (a secondary metabolite of plants), belonging to the family of hydroxycinnamic acids (HCAs). CFA is biosynthesized in plant tissues via the endogenous shikimate pathway, which is known to be responsible for the production of aromatic amino acids from glucose [1]. Phenylalanine is a precursor for the synthesis of CFA [2]. CFA can be found in many products consumed daily, such as coffee beans, green tea, tomatoes [3], potatoes [4], artichokes, carrots, lettuces, dark plums, cherries, gooseberries, blackcurrants, grapes [5], and herbs (basil, rosemary, oregano) (Figure 1) [6,7]. CFA and other phenolic compounds are involved in plants’ defense mechanism against insects, pathogens, animals (biotic stresses), and environmental conditions, such as excess water, drought, low and high temperatures, salinity, heavy metals, and ultraviolet radiation (abiotic stresses) [8,9]. Numerous in vitro and in vivo studies have shown that CFA has many biological properties, including anti-inflammatory [10,11,12], anticancer [1,13,14,15], antibacterial [16,17,18], antiviral [19,20], antidiabetic [21,22], hepatoprotective [23,24,25], and cardioprotective activity [26,27]. The presence of a catechol group with a chain of α,β-unsaturated carboxylic acids in the chemical structure of CFA affects its antioxidant properties. This antioxidant mechanism of action is based on the generation of an *o*-chinone group after the electron donation. Conjugation of the catechol group with the double side binding of *o*-quinone causes electron delocalization, increasing the stability of the *o*-quinone radical and the antiradical activity of CFA [28]. CFA can also form complexes with metals (e.g., with iron or copper), inhibiting the decomposition of peroxides, which limits the formation of free radicals and their negative impact on the organism. The excess of free radicals in the organism contributes to unfavorable changes/damage in the structure of proteins, lipids, carbohydrates, and DNA and triggers a number of diseases (e.g., atherosclerosis, asthma, cancer, diabetes, Alzheimer’s, and Parkinson’s diseases) [1,29,30].

**Figure 2:**
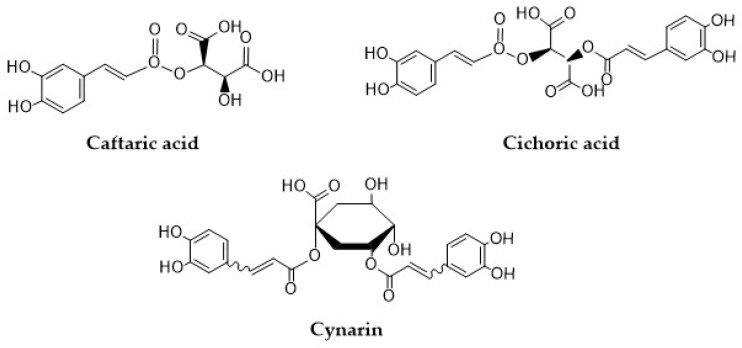
Structural formulas of caftaric acid, cichoric acid, and cynarin.

Compounds such as 2-caffeoyl-L-tartaric acid (caftaric acid, CTA), 2,3-dicaffeoyl-L-tartaric acid (cichoric acid, CA), and 1,5-dicaffeoylquinic acid (cynarin, CY) are natural conjugates of caffeic acid that have gained popularity in recent years because of their promising antioxidant properties (Figure 2). Structurally, these compounds are conjugates of tartaric acid with tartaric acid or quinic acid. Caftaric acid (CTA) is a major HCA, which can be found in all types of grape seeds and grape juice [31]. CTA is characterized by high bioavailability which has been confirmed in numerous in vitro studies [32]. In rats, CTA is quickly absorbed in the stomach, and can be detected in blood, kidneys, and in the brain, but not in the liver. CTA can also be found in urine as a conversion product—trans-fertaric acid [33,34]. Koriem and Soliman reported that CTA can alleviate methamphetamine (METH)-induced oxidative stress in male albino rats by preventing the accumulation of lipid peroxidation and by restoring liver superoxide dismutase (SOD), and glutathione peroxidase (GPx) activities [35]. In the study by Koriem et al. [33], CTA was found to exhibit an antioxidant effect by inhibiting LA (lead acetate)-induced oxidative damage in rat kidneys. In addition, CTA restored the LA-induced changes in p53 (tumor suppression gene) and bcl-2 (apoptosis inhibitory factor) gene expression to approximately normal levels [33]. CTA is one of the four major ingredients in sweet basil (*Ocimum basilicum* L.) extract (3.8 mg/g dry extract). In the work of Harnafi et al. [36], this extract exhibited a significant hypolipidemic effect by reducing plasma total cholesterol, triglycerides, and LDL (low-density lipoprotein) cholesterol, by 42%, 42%, and 86%, respectively. Moreover, the extract reduced the atherogenic index and LDL/HDL (high-density lipoprotein) cholesterol ratio by 88% and 94%, respectively [36].

Cichoric acid (CA) has several pro-health activities, including anti-diabetic [37], antiviral, anti-inflammatory [38], and anticancer activity. The study conducted by Tsai et al. [39] showed that CA may be a potential chemotherapeutic agent; the obtained results proved that CA significantly inhibited the activity of telomerase and induced apoptosis in human colon cancer cells (HCT-116) [39]. In the study by Xiao et al. [40], CA showed significant anti-obesity activity in vivo by lowering the serum lipid parameters and reducing the body weight of the tested mice [40]. Zhang et al. [41] reported the anti-hepatotoxic activity of CA isolated from the leaves of *Cichorium intybus*. A dose of 10–100 µg/mL CA reduced significantly the hepatitis B virus (HBV) surface and envelope antigen levels in HepG2.2.15 human hepatoblastoma cells, and produced the maximum inhibition rates of 79.94% and 76.41%, respectively. At a higher tested dose (50–100 µg/mL), CA significantly inhibited HBV DNA replication [41]. Moreover, the literature data indicate that CA is a potent inhibitor of the HIV-1 IN virus [42,43].

Cynarin (CY) is a major derivative of caffeoylquinic acids found in artichokes leaves and heads [44]. CY has been known to possess biological properties, including antioxidant [45], anti-diabetic [46], anti-atherosclerotic [47,48], hepatoprotective [49,50], anti-tumor [51,52], anti-HIV [53], choleretic [54], and immuno-suppressive activity [55]. In the study by Xia et al. [47], the treatment of HCASMC (human coronary artery smooth muscle cells) with CY led to a downregulation of iNOS (inducible nitric oxide synthase) mRNA and protein expression [47].

Caffeic acid is characterized by high biological activity. Due to the fact that in the plant world, it usually occurs in the form of combinations with other compounds, we wanted to answer the following questions:

(1) How does the electronic charge distribution within the caffeic acid moiety change after it creates the selected conjugates?
(2) How do these structural changes affect the activity of caffeic acid and its derivatives?

In this study, the physicochemical and biological properties of caffeic acid and its conjugates (caftaric acid, cichoric acid, and cynarin) were investigated. The molecular structures of compounds were studied using spectroscopic methods (FT-IR, Raman, UV-VIS, ^1^H, and ^13^C NMR), and quantum chemical calculations using the Gaussian 09W program. Antioxidant activity was evaluated by SOD-mimic activity and HO^•^ radical scavenging activity assays. The cytotoxicity of cichoric acid, caftaric acid, caffeic acid, and cynarin was tested on DLD-1 cell lines.

## 2. Materials and Methods

### 2.1. Materials

Cichoric acid, caftaric acid, caffeic acid, cynarin, KBr, XTT sodium salt (2,3-bis(2-methoxy-4-nitro-5-sulfophenyl)-2*H*-tetrazolium-5-carboxanilide inner salt), KO_2_, FeSO_4_∙7H_2_O, H_2_O_2_, and salicylic acid were purchased from Sigma-Aldrich Co. (St. Louis, MO, USA). Dimethyl sulfoxide (DMSO), hydrochloric acid (35%), methanol, and ethanol (analytical grade) were purchased from Chempur (Piekary Śląskie, Poland). All chemicals had an analytical purity and were used without further purification.

Dulbecco’s modified Eagle’s medium (DMEM) with 4.5 mg/mL (25 mM) of glucose with Glutamax, penicillin, streptomycin, trypsin–EDTA, FBS (fetal bovine serum) Gold, and PBS (phosphate-buffered saline) (without Ca and Mg) were provided by Gibco (San Diego, CA, USA). CellTiter-Glo™ 2.0 Assay was purchased from Promega (Madison, WI, USA).

### 2.2. Theoretical Studies

The optimal geometrical structures of CFA, CA, CTA, and CY and their frequencies of infrared vibrations were calculated using the B3LYP/6-311+G(d,p) method. The theoretical values of chemical shifts were calculated by the GIAO method in B3LYP/6-311+G(d,p) using DMSO as a solvent. The electronic charge distribution of the studied molecules was calculated using the NBO [56] and CHelpG [57] methods. The electrostatic potential (ESP) distribution maps were calculated using the CHelpG method [57]. The energy of HOMO and LUMO orbitals was calculated using the B3LYP/6-311+G(d,p) method. Based on the obtained HOMO and LUMO orbitals’ energy values, other reactivity descriptors, such as energy gap, ionization potential, electron affinity, electronegativity, chemical potential, hardness and softness, and electrophilicity index were calculated. The aromaticity indices were calculated from the length of the bonds in the aromatic ring of the optimized structures. All calculations were performed using the Gaussian 09W software package [58].

### 2.3. Spectroscopic Studies

The IR spectra of CFA, CA, CTA, and CY were performed by pressing the samples within KBr, and the ATR multi-reflection technique was also used. The spectra were recorded in the range of 4000–400 cm^−1^ by the use of an Alfa spectrometer (Bruker, Billerica, MA, USA). The Raman spectra were recorded using a Multi-Raman spectrophotometer (Bruker, Bremen, Germany) in the range of 4000–400 cm^−1^, with a laser power of 250 mW. The ^1^H and ^13^CNMR spectra of DMSO solution of the compounds were recorded with a Bruker Avance II 400 MHz unit at room temperature. Tetramethylsilane (TMS) was used as an internal reference. The UV-VIS spectra were recorded for the aqueous solution of the compounds at a concentration of 5.10^−5^ M in the range of 190–400 nm, using the UV/VIS/NIR Agilent Carry 5000 spectrophotometer (Santa Clara, CA, USA).

### 2.4. Antioxidant Assays

#### 2.4.1. SOD Activity

The use of the SOD-mimic in vitro test is relevant because of the importance of the SOD (superoxide dismutase) enzyme in the antiradical defense mechanism. The used method was based on the competitive reaction of the compounds, XTT dye, and KO_2_. The formation of orange XTT-formazane was the result of the interaction of XTT dye formed during the reaction of the superoxide anion radical. The SOD-mimic activity assay was performed based on the method described in [59]. Tested substances were dissolved in DMSO. The reaction mixture consisted of the following: 100 µL of tested substance in a concentration range of 0.05–0.4 mM, 2 mL of phosphate buffer (pH = 7.4; 0.01 M), 50 µL of XTT dye DMSO solution, and 100 µL of saturated KO_2_ in DMSO. Then, the samples were incubated for 30 min and the absorbance was measured at λ = 480 nm. Control samples without tested compounds were prepared in parallel. The inhibition level (I%) was calculated according to Formula (1):

I% = ((A_c_ − A_t_)/A_c_)) · 100% (1)

where A_c_ is the absorbance of the control sample and A_t_ is the absorbance of the tested sample.

#### 2.4.2. HO^•^ Radical Inhibition Activity

Hydroxyl radical inhibition assay was performed according to [60]. A total volume of 0.3 mL of FeSO_4_ (8 mM), 1 mL of salicylic acid ethanol solution (3 mM), and 0.25 mL of H_2_O_2_ (20 mM) were added to 1 mL of the tested compound in the concentration range of 0.1 mM–1 µM. Control sample consisted the same amounts of compounds but H_2_O was used instead of H_2_O_2_. Blank sample consisted of DMSO instead of tested compounds. All samples were incubated at 37 °C for 30 min, then 0.5 mL of H_2_O was added, and the absorbance was measured at λ = 510 nm. The inhibition level (I%) was calculated according to the following formula:

I% = (1 − ((A_c_ − A_t_)/A_b_)) · 100% (2)

where A_c_ is the absorbance of the control sample, A_t_ is the absorbance of the tested sample, and A_b_ is the absorbance of the blank sample.

The concentration of the tested compounds was plotted against the %I, and the IC_50_ values (antioxidant concentration that inhibited 50% of radicals) were calculated from the obtained scavenging curves.

### 2.5. Cytotoxic Study

The influence of CFA, CA, CTA, and CY was studied in relation to a colorectal adenocarcinoma DLD-1 cell line, which was obtained from the American Type Culture Collection (ATCC). DLD-1 cells were cultured in DMEM (Gibco) supplemented with 10% FBS (Gibco), penicillin (100 U/mL), and streptomycin (100 μg/mL) at 37 °C in a humified atmosphere of 5% CO_2_ in the air. The cells viability in tested cell lines was examined at the concentrations of 0.5 µM, 1 µM, 5 µM, 10 µM, 20 µM, 50 µM, 100 µM, 200 µM, 300 µM, and 500 µM for every studied compound.

#### 2.5.1. Chemical Treatment of Cells

CFA, CA, CTA, and CY were stored in a refrigerator at a temperature of 4 °C, and the stock solution was prepared by dissolving it in TrisHCl buffer. Compounds were added to the cultured cells for a final concentration in the range of 0.5 µM to 500 µM. The control cells were incubated without test compounds.

#### 2.5.2. Cichoric Acid, Caftaric Acid, Caffeic Acid, and Cynarin Cytotoxicity

CFA, CA, CTA, and CY cytotoxicity was measured with the use of CellTiter-Glo™ 2.0 Assay (Promega) according to manufacturer’s protocol. DLD-1 cells were seeded on a 96-well white plate at a density of 1 × 10^4^ cells/well, and after 24 h, the cells intended to be attached to the plate surface cells were treated with CFA, CA, CTA, and CY in a concentration range from 0.5 µM to 500 µM. After 24 h and 48 h, the cells were subjected to CellTiter-Glo™ 2.0 Assay (Promega). Luminescence was measured with a plate reader GloMax^®^-Multi Microplate Multimode Reader. The study was performed in triplicate to ensure consistent results were obtained.

#### 2.5.3. Statistical Analysis

All data are given as mean values ±SD (standard deviation). Differences between treatments and untreated control human cells were analyzed by one-way ANOVA, followed by Dunnett’s procedure for multiple comparisons. Significant effects are represented by *p* ≤ 0.05 (*), *p* ≤ 0.01 (**), *p* ≤ 0.001 (***).

## 3. Results

### 3.1. Theoretical Calculations

**Figure 3:**
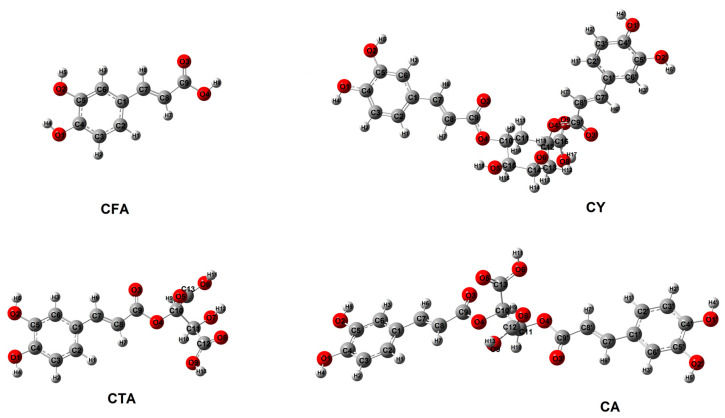
Structures of CFA, CA, CTA, and CY calculated in B3LYP/6-311++G(d,p).

The optimized structures of the analyzed compounds calculated using the B3LYP/6-311++G(d,p) method are shown in Figure 3. Table 1 presents the geometric indices of the studied compounds (energy, dipole moment, energy of HOMO and LUMO orbitals, values of aromaticity indices). The calculated values of the chemical shifts of protons and carbons from the ^1^H and ^13^C NMR spectra were analyzed and presented in the chapter “NMR study”, while the vibrational frequencies were theoretically analyzed and presented in the chapter “IR and Raman study”. The aromaticity indices were calculated on the basis of the bond lengths of the structures optimized by the DFT method. The calculated values of the π-electron systems make it possible to assess the aromaticity of the tested compounds, which is related to the stabilization of the aromatic ring and its reactivity. The aromatic ring of CFA exhibits a similar level or aromaticity in each of the presented structures. CY shows a slightly lower value of the HOMA index, the idea of which is based on the alternation of bonds in the aromatic ring, in relation to the other examined structures. The other values of the calculated indices for CTA, CA, and CY are at a similar level and slightly higher than the values for CFA. It can be concluded that in each of the tested systems, the ability to substitute in the aromatic ring will be similar and higher than in the CFA molecule. These compounds are characterized by high aromaticity; therefore, they will not be susceptible to substitution in the benzene ring. CFA is characterized by a slightly higher aromaticity index than its derivatives. The reactivity of the tested compounds is largely related to the ability of the hydroxyl groups to react with other chemicals.

**Figure 4:**
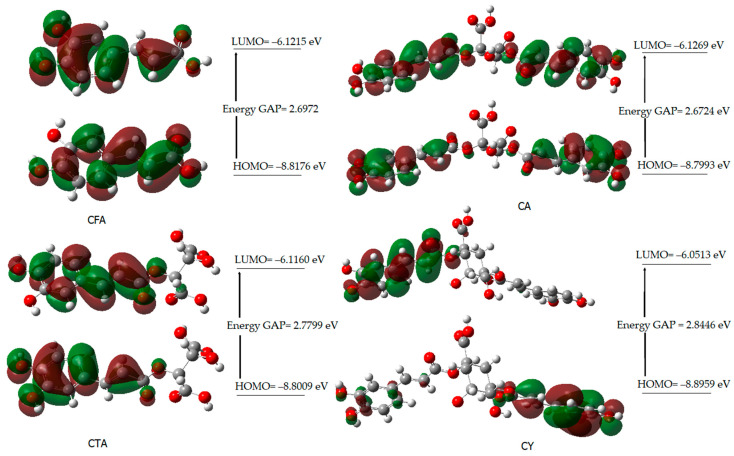
HOMO and LUMO molecular orbitals of CFA, CA, CTA, and CY.

Figure 4 shows the shapes of the orbitals for CFA, CA, CTA, and CY. The highest occupied molecular orbital (HOMO) and the lowest unoccupied molecular orbital (LUMO) play an important role in predicting the charge transfer in a molecule, chemical reactivity/ bioactivity, and compound stability [61]. The chemical potential $\mu =-\frac{\left(IP+EA\right)}{2}$ expresses the ability to detach and escape electrons from a stable system. Chemical hardness $\eta =\frac{\left(IP-EA\right)}{2}$ determines the resistance to the deformation of the electron cloud of a molecule under the influence of disturbances occurring in chemical reactions. Molecules with higher hardness are less susceptible to changes in the electronic charge distribution caused by the attachment of substituents, e.g., to the aromatic ring. The hardness of a molecule determines its resistance to changes in the distribution of electronic charge due to the disturbance of this charge. The HOMO-LUMO energy difference (energy GAP) determines the reactivity of the molecule. The larger the energy gap (HOMO-LUMO), the less reactive a given molecule is (the molecule is hard in local terms). The inverse of the hardness of a molecule is the softness described by the equation S = 1/2η.

The energy gap values (∆E) of the studied compounds decrease in the following series: CY > CA > CFA > CTA. This indicates a decrease in kinetic stability and an increase in their reactivity in the following order. CFA shows a lower reactivity than CY and CA. On the other hand, caftaric acid shows the lowest reactivity according to the calculated energy values of the HOMO and LUMO orbitals. The electrophilicity index (ω) provides information not only about the reactivity but also about the toxicity of the molecule. This parameter is related to energy stabilization when the system receives an additional electrostatic charge from the environment and quantifies the global electrophilic force of the molecule [62]. The electrophilicity index varies in the following series: CY > CFA > CA > CTA. This indicates that cynarin has the greatest electrophilic power.

### 3.2. NBO and ESP

**Figure 5:**
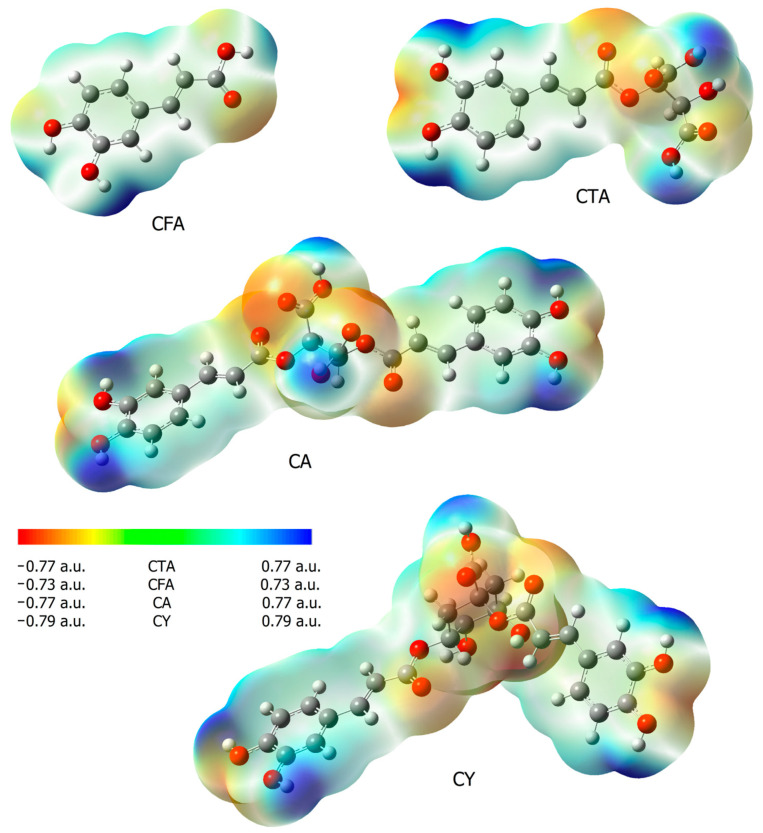
Electrostatic potential maps (calculated via the SCF in B3LYP/6-311++G(d,p)) for CFA, CA, CTA, and CY.

The reactivity of chemical compounds was also assessed on the basis of the calculated values of electron charges using the NBO method, and maps of electrostatic potential distribution. Table 2 shows the calculated values of the electron charges using the NBO and CHelp methods. Figure 5 shows the maps of the molecular electrostatic potential distribution (EPS). The electrostatic potential map shows the areas of a molecule related to its electrophilic (red) and nucleophilic (blue) reactivity (Figure 5). In CFA, it was observed that the oxygen atom of the carbonyl group was susceptible to electrophilic attack, while the areas of the molecule around the hydrogen atoms in the hydroxyl groups are characterized by increased susceptibility to nucleophilic attack. In the case of CTA and CA, additional centers susceptible to nucleophilic attack appear. These are the hydroxyl groups of the aromatic ring and the hydroxyl groups of a part of the molecule found in tartaric acid. The hydroxyl groups of CTA and CA are more susceptible to nucleophilic attack than in CFA. Fragments of caffeic acid included in the CY molecules show a similar distribution of electrophilic and nucleophilic susceptibility as in other molecules of the tested compounds. The hydroxyl groups of quinic acid present in the CY molecule show low nucleophilic activity.

Calculations of the electron charge distribution using the NBO method showed that the electron density around the atoms of the aromatic ring carbons labeled as C1, C2, and C3 increases in the CFA conjugates compared to the pure acid. In the case of the C4 carbon bonded to the hydroxyl group, in the series of compounds studied, the electron density around this atom is at a similar level. The electron density around the C5 atom is the highest in caffeic acid, while it takes on lower values in its conjugates. The electron densities around the aliphatic atoms C7 and C8 increase slightly in the conjugates with respect to CFA. In contrast, the electron density on the carboxyl group carbon C9 is highest in CFA compared to the conjugates of this acid. The changes in electron charge calculated by the ChelpG method do not coincide in every case with the results obtained by the NBO method.

The electron densities around the protons of the aromatic system H1, H2, and H3 calculated by the NBO method do not change significantly in the series of CFA derivatives. Larger changes were observed for the protons of the hydroxyl groups H4 and H5 (a significant decrease in the electron density at the conjugates with respect to CFA was noted). In the case of aliphatic protons H6 and H7, the observed changes in density around these atoms were also negligible.

### 3.3. IR and Raman Spectra

**Figure 6:**
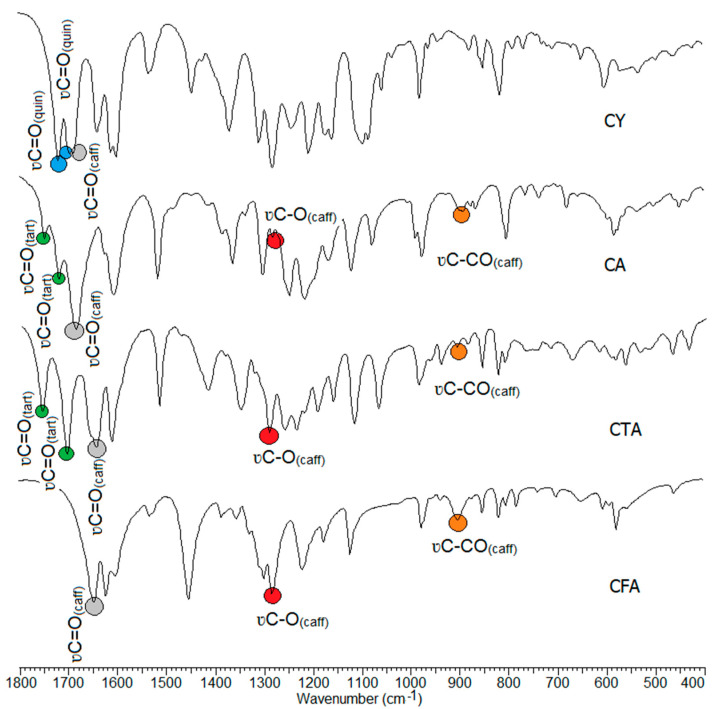
Experimental infrared (IR) spectra of CFA, CA, CTA, and CY.

The wavenumbers, intensities, and assignments of the selected bands occurring in the FT-IR (recorded in a KBr pellet, and using the ATR technique), the Raman spectra of the tested compounds, and the theoretical infrared vibrational frequencies are presented in Table 3. The FT-IR spectra of CFA, CA, CTA, and CY are presented in Figure 6. In the spectra of the studied compounds, there are characteristic bands derived from vibration bands of the caffeic, tartaric, and quinic acid carboxyl groups. The CFA spectra show characteristic bands assigned to the stretching vibrations of the carbonyl group ν(C=O) located at 1644 cm^−1^ (IR), and 1640 cm^−1^ (Raman). In the spectra of conjugates of CFA with tartaric acid, i.e., in CTA and CA, these bands are shifted toward higher values, i.e., up to 1647 cm^−1^ and 1682 cm^−1^ in the IR spectra, and 1648 cm^−1^, 1681 cm^−1^ in the Raman spectra. In the CY (caffeic acid and quinic acid conjugate) spectra, the wavenumbers of stretching ν(C=O) occurs at 1637 cm^−1^ (IR) and at 1638 cm^−1^ (Raman). In the CTA spectra, the bands assigned to the stretching vibrations of the tartaric acid carbonyl group are located at 1758 cm^−1^, 1707 cm^−1^ (IR), and at 1757 cm^−1^, 1706 cm^−1^ (Raman). These bands are slightly shifted to the values of 1748 cm^−1^, 1718 cm^−1^ in the IR spectrum of CA. CY is a conjugate of tartaric acid and quinic acid. Characteristic bands of stretching vibrations of the carbonyl group of quinic acid are observed in the CY spectra. They are located at 1716 cm^−1^, 1692 cm^−1^ in the IR spectra, and at 1715 cm^−1^, 1698 cm^−1^ in the Raman spectra. The bands resulting from stretching vibrations of C-OH bonds of the carboxyl group of CFA are observed in the spectra of all tested compounds.

A number of characteristic bands related to the vibrations of the aromatic system appear on the spectrum of CFA. The aromatic bands have been assigned according to Versanyi [63]. The formation of conjugates with tartaric or quinic acid causes changes in the spectrum of CFA. By observing the number of bands, position or intensity, it is possible to determine how the formation of conjugates affects the electron charge distribution in the ligand molecule. If the intensity of the bands decreases, the number of bands decreases or the wavenumber values of the aromatic system bands decrease, we are dealing with a disruption of the electron charge distribution in the molecule. Changes in the distribution of electronic charge are associated with a decrease in bond strength constants, which affects the position and intensity of bands on the IR spectra.

In the spectra of the studied conjugates, an increase in the intensity of some bands related to the vibrations of the aromatic system was observed compared to the spectrum of CFA. The wavenumbers of many bands shift toward higher values. These include the stretching bands ν(CH)_ar_ labeled 20a, the stretching bands ν(CC)_ar_ labeled 14, the out-of-plane bending bands γ(CH)_ar_ labeled 17a and 17b, and the deformation bands of the aromatic ring γ(CC)_ar_, def_ring ou_ labeled 16b. Bands that were not present in the spectra of CFA conjugates appear on the spectra of CFA. These are the bands labeled 9a and 4. There was an increase in the wavenumbers of the bands labeled 8a and 5 on the spectra of CTA and CA relative to those bands observed on the spectrum of CFA.

A decrease in the values of the wavenumbers of some bands on the spectra of CFA conjugates compared to the spectrum of CFA was observed. These are bands 19b, 18a, and 6a and 6b (Table 3) On the basis of the analysis of the IR and Raman spectra, it can be concluded that CTA, CA, and CY have higher aromaticity than CFA. However, it should be noted that the stabilization of the electron charge distribution of the aromatic ring of CY is lower in CTA and CA.

### 3.4. NMR Spectra

**Figure 7:**
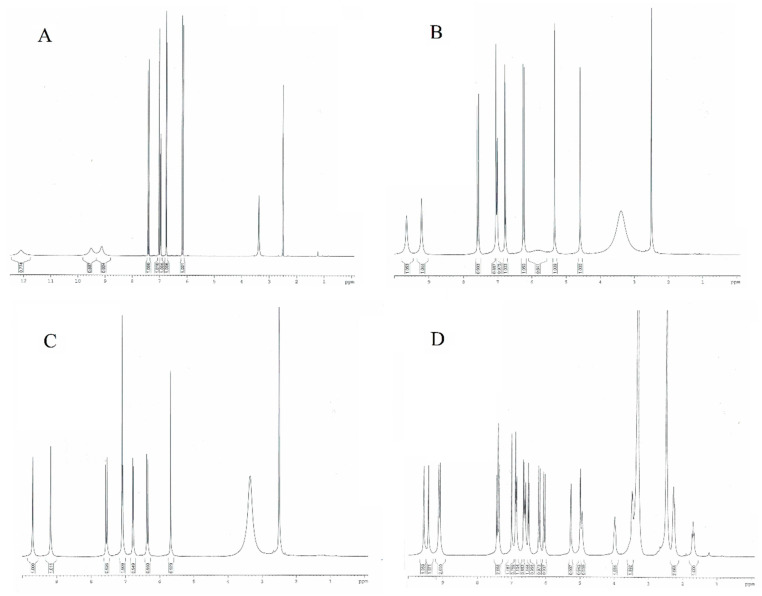
NMR spectra of (**A**) caffeic acid, (**B**) caftaric acid, (**C**) cichoric acid, and (**D**) cynarin.

As shown in Table 4 and Figure 7, the chemical shifts of the aromatic protons in the ^1^H NMR spectra of caffeic acid are 6.96, 6.75, and 7.02 ppm. The values of the signals of these protons in the spectra of CTA and CA shift toward higher values, indicating a decrease in the electron density around the nuclei of H1, H2, and H3 and an increase in the aromaticity of the CFA ring in its derivatives. In the case of CY, in which the CFA is linked to the quinic acid molecule, the opposite trend is observed. The values of the aromatic proton signals are lower as compared to CFA. The aromatic rings of CFA in CY show lower aromaticity than in pure CFA. Tartaric acid forming conjugates with CFA in derivatives (CTA, CA) has a stabilizing effect on the electron system of the aromatic ring of caffeic acid, whereas quinic acid (in CY) destabilizes the electron system. Also, calculations of the HOMA aromaticity index for the theoretically modeled structures showed that CY had a lower aromaticity than CFA, while in CTA and CA, the values of this index were higher. Around the protons of the hydroxyl groups H4 and H5, a decrease in electron density (an increase in signal shifts on the spectra) is observed in the conjugates of CFA compared to pure acid. This affects the reactivity of CFA derivatives in proton transfer-based free radical reactions. CFA conjugates are better free radical scavengers than pure CFA. The chemical shifts of the aliphatic protons H6 and H7 are lower in the conjugates compared to CFA. There is an increase in the electron density around the nuclei of these atoms when tartaric or quinic acid is attached to the CFA molecule.

The changes in the values of the chemical shifts of the carbons in the ^13^C NMR spectra of the studied compounds are related to the change in the electron density around the nuclei of the carbons. The values of the signals of the C1 and C2 aromatic ring carbons are lower in the CFA conjugates compared to the pure acid. This indicates an increase in electronic density around these atoms. In the case of the signals derived from the C4 and C5 carbons (attached to hydroxyl groups), there is an increase in the chemical shift values of these nuclei in the spectra of the CFA conjugates relative to the pure acid. In the case of the C6-labeled carbon, the values of the chemical shifts in all the structures studied are at similar levels. The electronic density around the aliphatic carbon labeled C8 in CFA conjugates is higher than in CFA as evidenced by the lower ppm value of the signal in the ^13^C NMR spectra of CTA, CA, and CY compared to CFA. In the case of the aliphatic carbon C7, the electronic density around this atomic nucleus is higher in CY than in CFA, while it has a lower value in CTA and CA. The carbon signals of the C9 carboxyl group take on lower values in the spectra of CY, CTA, and CA than in CFA, indicating an increase in the electronic density around this carbon after the formation of the CFA conjugates. The changes in electronic density around carbon atoms in the CFA conjugates relative to the pure acid, observed as changes in signal values on the ^13^C NMR spectra, follow a similar pattern to the data obtained by theoretical calculations carried out using the NBO method.

### 3.5. UV-VIS Spectra

**Figure 8:**
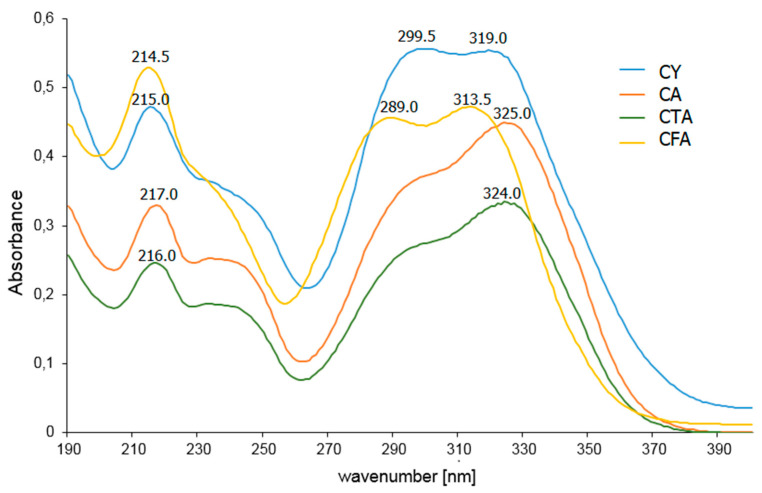
UV-VIS spectra of aqueous solutions of CFA, CA, CTA, and CY.

In the UV-VIS spectrum recorded for the aqueous solution of CFA, three bands associated with π→π* electron transitions are observed at the wavelengths λ_1_ = 214.5 nm, λ_2_ = 289.0 nm, and λ_3_ = 313.5 nm (Figure 8, Table 5). A bathochromic shift of the λ2 and λ3 bands is observed in the spectra of CFA conjugates. The bathochromic shift of the aromatic system bands in the spectrum indicates an increase in the aromaticity of the molecule. The λ_2_ band also undergoes a bathochromic shift in the UV-VIS spectrum of CY compared to that of CFA. This band flattens out on the spectral range of CA and CTA and the position of its maximum cannot be determined.

### 3.6. Antioxidant Activity

Theoretical studies (HOMO and LUMO energy calculations) show that CTA is a better electron acceptor molecule (better antioxidant) than CY. The lower the value of ∆E, the easier the electron enters the excited state in the molecule, and the better its antioxidant activity. Compounds with the lowest EA value have the highest electron transfer capacity, thus giving the highest SOD activity. According to the calculated theoretical parameters, CTA—with the highest energy of HOMO orbital (−8.52886 eV), and with the lowest values of ionization potential (8.52886 eV) and electronegativity (7.322444 eV)—should demonstrate the best antioxidant properties from the tested compounds. The antioxidant activity of CFA, CA, CTA, and CY has been investigated by hydroxyl radical (HO^•^) and superoxide radical (O_2_^•^) scavenging potentials (Table 6).

**Figure 9:**
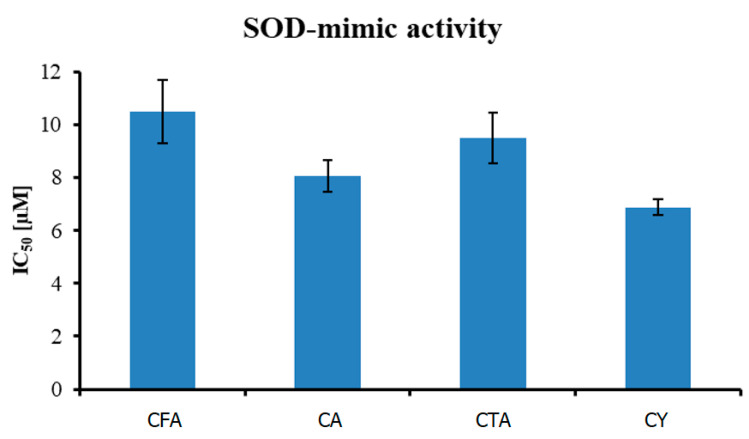
Antioxidant activity (measured using O_2_^•^ radical assay) of tested compounds.

As shown in Figure 9, out of four tested compounds, CY was the most active in the inhibition of superoxide anion formation, with an IC_50_ value equal to 6.880 ± 0.31 µM. CA and CTA were less effective, with IC_50_ 8.062 ± 0.59 and 9.510 ± 0.96 µM, respectively. CFA, with the least complex structure, had an IC_50_ equal to 10.491 ± 1.20 µM.

**Figure 10:**
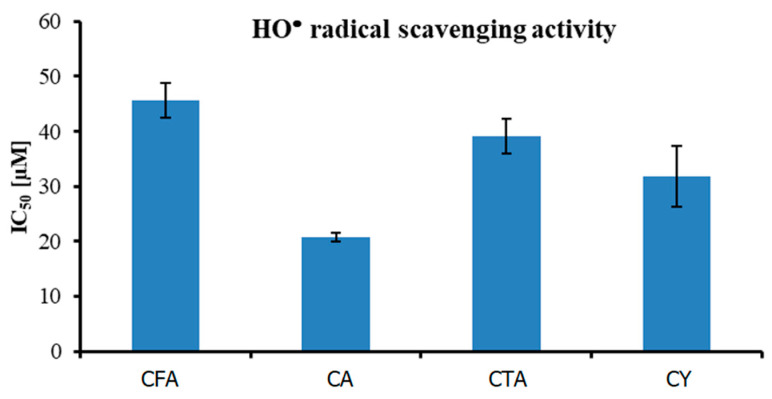
Antioxidant activity (measured using HO^•^ radical assay) of tested compounds.

The antioxidant activity against HO^•^ radical of four tested compounds is shown in Figure 10. IC_50_ value increased in the following order: CA (20.77 ± 0.86 µM) < CY (31.74 ± 5.51 µM) < CTA (39.18 ± 3.12 µM) < CFA (45.714 ± 3.15 µM). The obtained results prove the fact that CFA is the weakest antioxidant of the series. In all of the performed assays, CFA with the least complex structure was the weakest antioxidant. The presence of the two CFA moieties in the CA and CY molecules determines their high antioxidant properties. Substitution of the aromatic ring in ortho- or para-position can enhance the antioxidant activity because of the possible resonance structures leading to increased stability of the antioxidant radical formed upon the scavenging of other radicals. CFA has one ortho-dihydroxy phenyl group only, while CA and CTA are composed of two molecules of CFA. Moreover, among all the tested compounds, CY and CA have the highest number of hydroxyl groups in their structures, which may also affect their biological activity. The results of a previous study conducted by another research group are consistent with the results of the present work and showed that the antioxidant activity of CY and CA was superior to that of other tested compounds. Liu and coworkers [64], evaluated the HO^•^ free radical scavenging ability of CFA, CA, and CTA. They found that at compound concentrations of 500 µM, the degree of radical scavenging activity by CA was about 15.7% and 20.5% higher than that by CTA and CFA, respectively [64]. Many studies have shown that CA and CY have antibacterial, anti-inflammatory, and anti-HIV properties, which could be linked to their antioxidant activity [65,66].

### 3.7. Cytotoxicity

**Figure 11:**
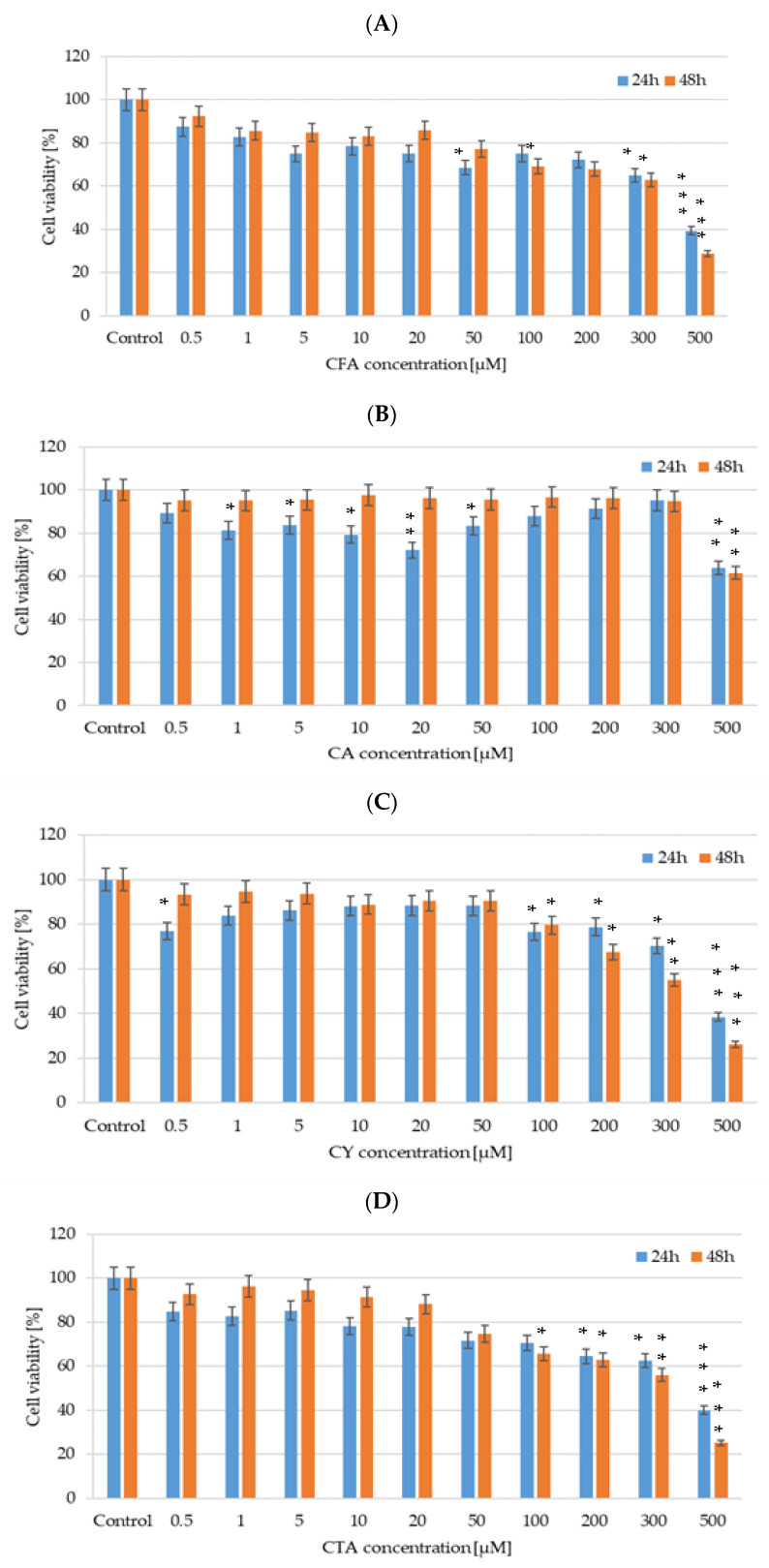
Cell viability results for DLD-1 cell lines exposed to different concentrations of (**A**) CFA, (**B**) CTA, (**C**) CA, and (**D**) CY for 24 h and 48 h, calculated as a percentage of control untreated cells. Each value on the graph is the mean of three independent experiments and error bars show the standard deviation (SD). * *p* < 0.05, ** *p* < 0.01, and *** *p* < 0.001 represent significant effects between treatments and control followed by a Dunnett’s test.

We evaluated the cytotoxicity of CFA, CA, CTA, and CY (0.5–500 µM) after 24 and 48 h of incubation on DLD-1 cancer cell lines using CellTiter-Glo™ 2.0 assay. As illustrated in Figure 11, all tested polyphenolic compounds exert a cytotoxic effect on an analyzed cell line. DLD-1 colorectal adenocarcinoma cells treated with CA exhibited a statistically significant decrease in relative cell viability even in low concentrations such as 10 µM and 20 µM after 24 h of incubation, but an IC50 value was obtained for the highest analyzed concentration—500 µM. In the case of CY activity in DLD-1 cells, the most inhibitory effect on cell viability was noticed for 500 µM of CY, causing a decrease higher than 70%. An inhibition of DLD-1 cell viability by about 50% was observed in a 300 µM CY 48-hour treatment. In the case of CFA influence on DLD-1 cells, a tendency in cell viability decreasing simultaneously with an increase in the studied compound concentration was noticed. Similar results were obtained for CTA.

## 4. Conclusions

The formation of the conjugates of caffeic acid (CFA) causes changes in the electronic charge distribution within the CFA molecule, which in turn affects the biological activity of molecules. Spectroscopic studies (FT-IR, Raman, UV-VIS, ^1^H NMR, ^13^C NMR) showed higher aromaticity of conjugates of CFA (i.e., CFA, CA, and CY) compared to the unconjugated molecule of CFA. CFA, CA, and CY are characterized by an increased stability of the electronic arrangement in the aromatic ring compared to CFA. Theoretical calculations (NBO, electrostatic potential map) and experimental studies (^1^H NMR, ^13^C NMR) showed that the electronic density around the protons of the hydroxyl groups in the conjugate molecules (CTA, CA, and CY) is higher than in free CFA, which causes the increase in the antioxidant capacity of these molecules. The antioxidant assays showed that CFA conjugates possessed higher antiradical activity toward superoxide radical O_2_^•^ and the hydroxyl radical HO^•^ than CFA. Theoretical calculations (including the calculation of the HOMO and LUMO orbitals’ energy confirm these findings. CFA and its conjugates showed cytotoxic properties against DLD-1 cell lines. CA showed the best cytotoxic effect against DLD-1 cells and reduced cell viability at the lowest concentrations used in the study. Slightly weaker cytotoxic potential was exhibited by CY, CTA, and CFA.

## Figures and Tables

**Table 1:** Energy parameters and aromaticity for CFA, CA, CTA, and CY calculated in B3LYP/6-311++G(d,p).

	CFA	CTA	CA	CY
Energy [hartree]	−648.8686	−1179.9939	−1752.3902	−1869.9097
Energy [eV]	−17,656.6036	−32,109.2506	−47,684.9381	−50,882.8000
Dipole moment [D]	2.1285	3.0949	1.6335	3.7528
HOMO [eV]	−8.8187	−8.5289	−8.7993	−8.8959
LUMO [eV]	−6.2477	−6.1160	−6.1269	−6.0513
Energy gap [eV]	2.5709	2.4128	2.6724	2.8446
Ionization potential, I = −E_HOMO_	8.8187	8.5289	8.7993	8.8959
Electron affinity, A = −E_LUMO_	6.2477	6.1160	6.1269	6.0513
Electronegativity, χ	7.5332	7.3224	7.4631	7.4736
Chemical potential, μ	−6.2477	−6.1160	−6.1269	−6.0513
Chemical hardness, η	1.2855	1.2064	1.3362	1.4223
Chemical softness, S	0.3890	0.4145	0.3742	0.3515
Electrophilicity index, ω	100.3540	90.2539	100.3204	104.1770
Aromaticity indices				
Aj	0.991	0.993	0.993	0.993
BAC	0.875	0.885	0.884	0.886
HOMA	0.956	0.958	0.958	0.939
EN	0.020	0.025	0.025	0.045
GEO	0.020	0.016	0.016	0.016
I6	92.51	93.30	93.29	93.39

**Table 2:** Electron density calculated using NBO and ChelpG methods (B3LYP/6-311++G(d,p) for CFA and their conjugates.

	CFA		CTA		CA		CY	
	NBO	CHelpG	NBO	CHelpG	NBO	CHelpG	NBO	CHelpG
H1	0.207	0.090	0.207	0.103	0.208	0.087	0.207	0.127
H2	0.222	0.155	0.204	0.217	0.204	0.237	0.205	0.150
H3	0.205	0.182	0.203	0.262	0.202	0.224	0.203	0.199
H4	0.488	0.362	0.469	0.298	0.469	0.345	0.469	0.375
H5	0.482	0.378	0.469	0.302	0.468	0.315	0.469	0.369
H6	0.215	0.098	0.214	0.035	0.214	0.114	0.216	0.138
H7	0.211	0.207	0.213	0.183	0.214	0.093	0.208	0.092
H8	0.481	0.359	-	-	-	-	-	-
O1	−0.662	−0.513	−0.649	−0.452	−0.649	−0.554	−0.650	−0.529
O2	−0.705	−0.515	−0.657	−0.483	−0.657	−0.447	−0.658	−0.610
O3	−0.618	−0.599	−0.603	−0.582	−0.605	−0.571	−0.611	−0.539
O4	−0.689	−0.589	−0.564	−0.602	−0.555	−0.534	−0.585	−0.478
C1	−0.107	0.226	−0.112	0.216	−0.118	0.328	−0.120	0.259
C2	−0.168	−0.195	−0.171	−0.163	−0.171	−0.070	−0.173	−0.096
C3	−0.231	−0.195	−0.259	−0.382	−0.259	−0.556	−0.260	−0.330
C4	0.295	0.324	0.294	0.325	0.294	0.493	0.294	0.251
C5	0.249	0.231	0.275	0.442	0.271	0.354	0.272	0.608
C6	−0.225	−0.408	−0.225	−0.604	−0.214	−0.525	−0.215	−0.582
C7	−0.095	−0.042	−0.073	0.098	−0.075	−0.230	−0.083	−0.178
C8	−0.316	−0.419	−0.315	−0.419	−0.314	−0.145	−0.305	−0.264
C9	0.760	0.854	0.778	0.904	0.777	0.784	0.771	0.743

**Table 3:** The wavenumbers [cm^−1^], intensities, and assignments of bands observed in the experimental FT-IR (KBr and ATR) and FT-Raman spectra and theoretical FT-Raman spectra (calculated by DFT-B3LYP/6-311++G(d,p) method) of CFA, CA, CTA, and CY.

CFA					CTA					CA					CY					Assignments	[63]
IR_KBr_	IR_ATR_	Raman	DFT	Int.	IR_KBr_	IR_ATR_	Raman	DFT	Int.	IR_KBr_	IR_ATR_	Raman	DFT	Int.	IR_KBr_	IR_ATR_	Raman	DFT	Int.		
3433 s	3434 m		3850	89.9	3487 s	3485 m		3835	64.4	3412 s	3408 w		3835	109.2	3398 vs	3399 m		3829	61.7	νOH_ar_	
3236 s	3228 w		3783	160.6	3239 m	3229 w		3832	122.2	3359 s	3352 w	3352 w	3831	240.4	3234 w			3825	119.7	νOH_ar_	
			3770	119.9				3742	71.3				3755	92.2				3813	94.7	νOH_COOH_/νOH_chin_	
3060 m			3203	6.4	3063 w	3062 vw	3069 w	3202	5.4	3061 w			3203	5.9	3041 m		3043 w	3206	2.6	ν(CH)_ar_ + ν(CH)_C=C_	2
3025 m			3186	4.9				3194	1.7				3195	4.0	3022 w		3024 w	3172	16.3	ν(CH)_ar_ + ν(CH)_C=C_	20b
2924 m			3153	9.5	2953 w		2961 w	3155	15.4	2954 w		2963 w	3154	24.5	2925 m		2931 w	3169	12.4	ν(CH)_ar_	20a
					1758 s	1758 m	1757 vw	1842	252.1	1748 m	1746 w		1835	236.0	1716 vs	1716 s	1715 w			νC=O_tart_ /νC=O_chin_	
					1707 vs	1707 s	1706 w	1812	332.8	1718 s	1716 m		1824	352.5	1692 vs	1692 m	1698 w	1822	254.4	νC=O_tart_ /νC=O_chin_	
1644 vs	1643 s	1640 m	1775	351.9	1647 vs	1646 s	1648 m	1771	276.6	1682 vs	1679 vs	1681 s	1765	495.5	1637 s	1639 m	1638 m	1769	292.2	νC=O_caff_, ν(C=C)_C=C_	
1618 vs	1619 s	1612 vs	1679	193.4				1678	228.2	1624 m		1627 s	1678	474.8	1609 s	1609 s	1609 s	1685	318.2	νC=C_C=C_	
1600 s	1600 s	1594 m	1643	163.8	1616 vs	1616 s	1617 vs	1639	438.2	1606 s	1604 m	1609 vs	1638	901.8	1598 vs	1598 s	1599 vs	1646	621.1	ν(CC)_ar_, ν(C=C)_C=C_	8a
1530 m	1531 w	1531 w	1631	310.4	1518 s	1518 s	1518 vw	1633	57.3	1515 s	1515 m	1515 w	1632	106.3	1532 m	1534 m		1641	73.2	νCC_ar_, νC=C_C=C_, βCH_ar_,	8b
			1557	187.9	1473 w	1474 w	1476 w	1560	130.3	1484 w	1489 w	1482 w	1559	266.5		1508 w		1566	206.5	β(CC)_ar_, β(CH)_ar_, ν(CC)_ar_,	19a
1450 vs	1449 vs	1450 vw	1472	13.1	1418 m	1418 m		1466	46.9	1447 w			1466	94.7	1445 m	1446 m		1471	54.5	ν(CC), β(CH)_tart_	19b
1384 w	1375 w				1384 w			1415	36.5	1384 m	1385 w									β(CH)_ar,_ β(CH)_tart_	
1353 m	1353 w	1352 w	1404	16.6	1353 s	1352 s	1351 w	1382	115.4	1362 s	1362 m	1363 w	1381	340.7	1367 s	1364 s	1365 w	1393	181.4	ν(CC)_ar_, βOH_ar_, β(CH)_tart_	14
					1323 m	1324 w	1325 vw	1378	83.4	1337 w	1336 w	1342 vw	1421	42.8				1378	9.1	β(CH)_tart_	
1296 s	1296 s	1298 vs	1381	93.3									1381	340.7	1307 s	1306 m	1308 w	1364	22.8	βOH_ar_, νCC_ar_, β(CH)_C=C_	
			1354	25.1	1293 s	1294 s	1286 m	1347	10.2	1302 s	1300 m	1303 m	1362	9.9				1337	147.8	def_ring_, νCC_ar_, βCH_ar_, βOH_ar_	
1280 vs	1277 vs	1285 m	1312	260.5	1262 s	1258 s	1265 w	1306	217.5	1280 m	1281 w	1271 w	1333	216.7	1279 vs	1277 vs	1279 m	1313	397.3	ν(C−OH)_caff_, βCH_ar_, βCH_tart_	
					1238 s	1238 vs	1234 w	1305	43.0	1246 s	1246 s	1248 m	1321	51.2	1241 s	1239 s		1290	86.6	βCH_tart_	
			1293	94.0				1285	124.1				1305	440.2				1268	37.6	βCH_ar_, βCH_tart_, ν(C−O)	
1217 s	1217 s		1215	127.9				1209	54.1				1209	106.5				1214	71.5	βOH, βCH_ar_	
			1191	33.7	1222 s	1224 m	1215 vw	1193	3.5	1215 s	1203 s	1210 w	1193	35.1	1205 s	1202 vs	1193 m	1193	3.6	def_ring_, βCH_ar_, β(CH)_C=C_	9a
					1195 s	1195 s	1195 w	1172	150.6											def_ring_, βOH_tart_	
1174 m	1174 m	1186 m	1171	126.1	1163 m	1164 m	1164 w	1151	261.3	1166 m	1168 m	1169 w	1190	473.4	1171 s	1170 s	1168 w	1189	214.4	βCH_ar_, β(CH)_C=C_	18a
								1140	1351.9						1157 s	1157 s		1132	871.5	βCOH, βCH_tart,_ βOH_tart_	
1120 m	1120 m	1107 w	1121	55.3	1120 s	1122 s	1118 w	1117	174.9	1120 s	1121 m	1125 w	1116	334.2			1111 w	1117	192.9	βCH_ar_, βOH_ar_	18b
			1020	31.0	1069 s	1069 s	1071 vw	1015	28.4	1077 m	1076 m		1015	51.0	1082 s					βOH_caff,_ γ(CH)_C=C_	
			968	16.0	989 m	989 m	989 vw	983	20.2	988 m	989 w	981 vw	981	41.7						def_ring_, β(CH)_C=C_, νCC_tart_	
974 m	974 m	975 w	966	6.9	966 m	964 w		975	6.2	975 m	972 m	974 vw	969	29.6	979 m	979 m	978 w	974	1.9	γ(CH)_C=C,_ γ(CH)_ar_	17b
936 w	936 w	954 vw	950	1.7	943 w	943 m		925	1.1	926 w			925	1.9	940 w		962 w	928	1.3	γ(CH)_ar_	17a
900 m	898 m				909 w	909 w	910 w			890 w	896 w	897 vw						919	9.7	ν(CCO)_caff_, def_ring_ /def_chin_	
872 vw					888 w	888 w				876 w	876 w	878 w						887	4.3	β(CH)_C=C_, def_ring_ /def_chin_	
849 m	850 m	852 vw	890	7.2	858 m	858 m	858 w	888	6.5	866 w	866 w	862 w	888	12.3	848 m	848 m		884	3.2	γCH_ar_, γ(CH)_C=C_	5
817 m	816 m		852	51.9	826 m	826 s	824 vw	851	37.2				852	7.2	814 m	824 m		854	39.3	γCH_ar_, γ(CH)_C=C_	
801 w	803 m	802 w	828	15.2	811 m	813 m	813 w	806	26.5	803 m	803 m	808 w	807	47.5		814 m		808	30.2	γCH_ar_,	10a
780 w	780 m	779 vw	808	18.4	768 w	762 m		805	27.2	764 w	766 w	765 vw	788	49.6	787 w	766 w	771 w	805	20.0	def_ring_, βOH, β(CH)_C=C_	12
736 w			782	18.4	749 w	749 w	751 vw	786	21.1	736 w	736 w	730 w	746	5.9	740 w			797	10.7	def_ring_, ν(CC)_ar_	
					718 w	718 m	721 vw	743	9.4	715 vw			738	5.2				769	52.8	γ(C=O)_tart_, γ(C=O)_chin_	
699 w	700 w		751	10.5		697 w		737	13.9	698 vw	698 vw	699 w	736	2.2	707 w	717 w	729 w	741	1.4	γ(C=O)_caff_	
		686 vw	710	4.0	674 m	676 m		699	0.3	679 w			706	0.3	668 w			682	0.1	def_ring_,	4
648 w	649 m		657	21.7		642 w		658	8.8	658 w	661 w	663 vw	673	20.6	647 w	649 m		655	18.3	β(C=O)	
	616 w		610	67.3	617 m	616 m	596 vw	598	22.4	596 w		597 vw	598	30.4				613	82.6	def_ringou_, γOH	16a
603 w	603 m	601 w	599	18.2	595 w			596	2.7	583 m		585 w	583	1.8	600 m			590	23.9	def_ring_	6a
576 m			571	38.0	565 w		567 vw	578	41.9				581	79.9	568 m			578	26.3	def_ring_	6b
			564	45.1	518 w			549	38.8	502 w			553	0.6	531 m			502	6.9	γOH_caff_, γOH_tart_	
458 vw		460 vw	458	8.9	469 w			452	4.3	450 w		484 vw	454	7.3	460 w		458 w	455	4.5	γ(CC)_ar_, def_ringou_	16b
		446 w	434	67.7	435 w			389	91.7	431 w		436 vw	432	7.8	421 w		429 w			γOH_ar_, γOH_tart_	

* The intensities (Int.) of the bands were assigned as follows: wv—very weak, w—weak, m—medium, s—strong, vs—very strong and ** The types of vibrations were assigned as follows: ν—stretching vibrations, β—in-plane bending modes, γ—out-of-plane bending modes, def.—deforming vibrations, def_ring_—deforming vibrations of the aromatic ring, _ou_—out-of-plane bending vibrations, _caff_—vibrations of the atoms of caffeic acic, _tart_—vibrations of the atoms of tartaric acid, _al_—aliphatic atoms, _ar_—aromatic system atoms.

**Table 4:** Chemical shifts observed in NMR spectra of CFA, CA, CTA, and CY.

	CA		CTA		CA		CY	
	Exp.	Calc.	Exp.	Calc.	Exp.	Calc.	Exp.	Calc.
H1	6.96	6.67	7.58	7.72	7.54	7.63	6.63	7.87, 8.00
H2	6.75	6.47	6.78	7.03	7.10	6.90	6.51	7.26
H3	7.02	7.53	7.05	7.05	7.08	6.90	7.00	7.21
H4	9.51	3.90	9.65	4.93	9.70	4.66	9.56	5.28
H5	9.12	4.97	9.21	4.61	9.18	4.31	9.42	5.06
H6	7.41	7.71	7.02	8.03	6.78	7.84	6.20	8.23
H7	6.17	6.17	6.24	6.70	6.38	6.57	6.06	6.86, 6.72
H8	1.10	5.25	-	-	-	-	-	
C1	125.71	132.75	125.37	133.41	125.23	131.83	125.25	114.97
C2	121.16	126.72	121.54	125.35	115.81	123.80	120.42	106.43
C3	115.13	121.63	114.93	122.38	115.26	120.58	115.85	107.39
C4	145.57	155.83	146.48	155.49	147.04	153.81	148.27	134.73
C5	144.59	149.80	145.65	150.42	145.62	149.56	145.57	130.55
C6	115.76	124.91	115.87	126.68	121.74	125.48	115.53	108.94
C7	148.14	157.11	148.72	156.63	148.87	155.96	145.33	135.54
C8	114.65	116.09	112.92	116.92	112.37	114.05	114.19	99.64
C9	167.91	175.12	165.67	174.00	165.53	172.88	165.64	155.24

**Table 5:** The wavelengths of maximum absorbance from the UV-VIS spectra of CFA, CA, CTA, and CY.

	CFA	CTA	CA	CY
λmax_1_ [nm]	214.5	216.5	217.0	215.0
λmax_2_ [nm]	289.0	-	-	299.5
λmax_3_ [nm]	313.5	324.0	325.0	319.0

**Table 6:** Results of the various antioxidant activity tests of tested compounds.

IC_50_:	HO^•^ [µM]	O_2_^•^ [µM]
**Caffeic acid**	45.714 ± 3.15	10.491 ± 1.20
**Cichoric acid**	20.768 ± 0.86	8.062 ± 0.59
**Caftaric acid**	39.180 ± 3.12	9.510 ± 0.96
**Cynarin**	31.741 ± 5.51	6.880 ± 0.31

## Data Availability

The data presented in this study are available on request from the corresponding author.
